# Innate immune properties of selected human neuropeptides against *Moraxella catarrhalis* and nontypeable *Haemophilus influenzae*

**DOI:** 10.1186/1471-2172-13-24

**Published:** 2012-05-02

**Authors:** Daria Augustyniak, Adam Jankowski, Paweł Mackiewicz, Agnieszka Skowyra, Jan Gutowicz, Zuzanna Drulis-Kawa

**Affiliations:** 1 Department of Pathogen Biology and Immunology, Institute of Genetics and Microbiology, University of Wroclaw, Przybyszewskiego 63/77, 51-148, Wroclaw, Poland; 2 Department and Clinics of Pediatrics, Immunology and Rheumatology of Developmental Age, Medical University of Wroclaw, Kasprowicza 64/66, 51-137, Wroclaw, Poland; 3 Department of Genomics, Faculty of Biotechnology, University of Wroclaw, Przybyszewskiego 63/77, 51-148, Wroclaw, Poland; 4 Depertment of Physico-Chemistry of Microorganisms, Institute of Genetics and Microbiology, University of Wroclaw, Przybyszewskiego 63/77, 51-148, Wroclaw, Poland

**Keywords:** Neuropeptide Y, Substance P, CGRP, Somatostatin, Killing, Permeabilization, Phagocytosis, Immunomodulation, *Moraxella catarrhalis*, *Haemophilus influenzae*

## Abstract

**Background:**

Considerable evidence supports the concept of active communication between the nervous and immune systems. One class of such communicators are the neuropeptides (NPs). Recent reports have highlighted the antimicrobial activity of neuropeptides, placing them among the integral components of innate immune defense. This study examined the action of four human neuropeptides: calcitonin gene-related peptide (CGRP), neuropeptide Y (NPY), substance P (SP) and somatostatin (SOM), which are accessible in the upper respiratory tract, against two human-specific respiratory pathogens. We studied: (i) neuropeptide-mediated direct antibacterial activity exerted against *Moraxella catarrhalis* and nontypeable *Haemophilus influenzae,* and (ii) indirect immunomodulatory role of these neuropeptides in the neutrophil-mediated phagocytosis of indicated pathogens.

**Results:**

We found that 100 micromolar concentrations of CGRP, NPY, SP, and SOM effectively permeabilized bacterial membranes and showed (except SOM) bactericidal activity against both pathogens. SOM acted only bacteriostatically. However the killing efficacy was dependent on the bactericidal assay used. The rank order of killing NP effect was: NPY ≥ CGRP > SP >> SOM and correlated with their potency to permeabilize bacterial membranes. The killing and permeabilization activity of the analyzed NPs showed significant correlation with several physicochemical properties and amino acid composition of the neuropeptides. *M. catarrhalis* was more sensitive to neuropeptides than nontypeable *H. influenzae*.

The immunomodulatory bimodal effect of physiological concentrations of CGRP, NPY, and SP on the phagocytic function of human neutrophils against *M. catarrhalis* and *H. influenzae* was observed both in the ingestion (pathogen uptake) and reactive oxygen species generation stages. This effect was also dependent on the distinct type of pathogen recognition (opsonic versus nonopsonic).

**Conclusions:**

The present results indicate that neuropeptides such as CGRP, NPY, and SP can effectively participate in the direct and indirect elimination of human-specific respiratory pathogens. Because the studied NPs show both direct and indirect modulating antimicrobial potency, they seem to be important molecules involved in the innate host defense against *M. catarrhalis* and nontypeable *H. influenzae*.

## Background

Central nervous system-mediated regulation of immunity occurs through systemic, regional and local routes. The peripheral nervous system provides a first line of defense at local sites through the release of neuropeptides (peptide neurotransmitters) from sensory nerves [1]. Sensory neuropeptides such as calcitonin gene-related peptide (CGRP) and substance P (SP) as well as autonomic neuropeptide Y (NPY) and somatostatin (SOM) are widely distributed throughout the nervous system [2]. Numerous reports highlighted their role as bioactive molecules that exert immunomodulatory effects on innate and adaptive immune responses. The majority of the responses involve: (i) modulation of cytokine release by lymphocytes, monocytes/macrophages, and dendritic cells [3-5], (ii) regulation of lymphocyte and dendritic cells adhesion and activation [3,6], (iii) modulation of phagocytosis process [7,8], and (iv) modulation of inflammatory response [9].

Unlike the lower respiratory tract, the upper human airways are richly innervated [10]. SP and CGRP-containing nerves are present beneath and within the epithelium, around blood vessels and sub-mucosal glands and within the bronchial smooth-muscle layer [11]. Numerous nerves (with SP and CGRP) are located within the mucosal lining of the middle ear cavity [12]. Nasal mucosa is densely innervated by fibers containing NPY, SP, and CGRP [13,14]. The potential role of SP and CGRP in the pathogenesis of microbial-induced/associated airway diseases such as *otitis media* with effusion or chronic obstructive pulmonary disease has been postulated [15,16]. Airway lymphoid organs such as lymph nodes and tonsils receive both autonomic/sympathetic and sensory peptidergic innervations [10]. Interestingly, NPY, SP, and CGRP can be synthesized and released also from the immune cells such as lymphocytes or monocytes/macrophages [17-19]. Due to their cationic charge at neutral pH, low molecular mass (<10 kDa), and amphipathic properties, neuropeptides interact with the negatively charged surface of the microbial membrane resulting in membrane disruption and direct killing of the target cell [20]. Similarities between the NPs and the endogenous antimicrobial peptides led to the discovery of NPs antimicrobial activity directed against various bacteria and fungi [21-23], and place neuropeptides among factors that are capable of forming the local barriers of defense against pathogens.

*Moraxella catarrhalis* and nontypeable *Haemophilus influenzae* are important human-restricted respiratory tract pathogens that may occasionally cause invasive diseases [24,25]. They are both predominant etiological factors of *otitis media* and form biofilms in children with recurrent or chronic *otitis media*[26]. They both commonly colonize the lower airways of patients with chronic obstructive pulmonary disease, thus contributing to its pathogenesis [27,28]. Both species asymptomatically colonize the pharyngeal mucosa, a *port of pathogen entry*, and are present in nasopharyngeal secretions [29,30]. Colonization of host mucosal surfaces is the first and necessary step in the infectious process. Since the mucosal surfaces of the upper respiratory tract are richly innervated, both species are potentially exposed to the action of released neuropeptides.

This study was designed to elucidate two aspects of the antibacterial properties of neuropeptides CGRP, NPY, SP, and partly SOM: (i) the direct activity against *Moraxella catarrhalis* and nontypeable *Haemophilus influenzae*, (ii) the indirect action through the modulation of neutrophil phagocytosis by determination of bacterial uptake and respiratory burst response. We demonstrated temporary bactericidal activity of NPs as well as their opposite immunomodulatory action with reference to both studied critical phagocytic functions of neutrophil response against human-restricted respiratory pathogens.

## Methods

### Neuropeptides, reagents, and media

Calcitonin gene related peptide (α-CGRP), neuropeptide Y (NPY), substance P (SP), and somatostatin (SOM) were purchased from TOCRIS bioscences (Ellisville, MO, USA), NPY amino acid fragment NPY_18-36_ (NPYf) was bought from Sigma Chemicals (St Louis, MO, USA). All neuropeptides were dissolved in 0.01% (v/v) acetic acid containing 0.1% human serum albumin (Sigma) and stored at – 20°C in working stock solutions at concentration of 10^−3^ M. Other reagents and supplies such as acridine orange, hemin, luminol, β-nicotinamide adenine dinucleotidem (NAD), *o*-nitrophenyl-β-D-galactopyranosid (ONPG), phorbol myristate acetate (PMA), polymyxin B sulfate salt, and trypan blue were from Sigma (Steinheim, Germany). Other reagents were: ficoll 1.115 g/ml (Aqua-Med, Poland), FITC (Thermo Scientific, Rokford, USA), fMLF (Fluka, Switzerland), TSB – tripticasein soy broth (BIOCORP, Poland), BHI – brain heart infusion broth (Merck, Germany), HAEM chocolate plates (OXOID, Germany), agar-agar (Fluka, Spain), NaPB–1% BHI, sodium phosphate buffer (pH 7.4) containing 1% BHI (w/v); HBSS buffer with Ca^++^and Mg^++^ (PAA, Austria), heat inactivated fetal bovine serum (HiFBS), GIBCO BRL.

### Bacterial strains

*M. catarrhalis* ATCC 25238 and *H. influenzae* ATCC 49247 strains were used. Two clinical nasopharyngeal isolates of *Moraxella catarrhalis* (Mc5, Mc6) and nontypeable *Haemophilus influenzae* (NTHi3, NTHi6) were also included in some experiments. In ONPG assay, *Escherichia coli ML*-35p a lactose permease-deficient strain with constitutive cytoplasmic β-galactosidase activity (from dr Timothy Starner) was used. The clinical isolates were obtained from Laboratory of Bacteriology of the Silesian Centre of Pediatrics in Wroclaw, Poland. The final identification of *M. catarrhalis* was made using a standardized commercial identification system API-NH (Bio-Merieux, France). *H. influenzae* strains were identified by both X and V factor requirements, growth on chocolate agar but not on blood agar. The absence of *bexA* gene was confirmed by PCR capsular genotyping using *H. influenzae* type b as positive control [31]. NTHi strains were cultivated on HAEM chocolate agar plates or in BHI broth supplemented with hemin and NAD at final concentrations of 15 μg/ml. *M. catarrhalis* strains were grown in BHI. *E. coli* ML-35p was maintained on TSB. All Mc and NTHi strains were grown at 37°C with 5% CO_2_. The strains were stored in relevant medium containing 16% glycerol at −70°C.

### Antimicrobial assays

To evaluate antimicrobial properties of purified neuropeptides two assays were used: (i) a radial diffusion assay [32] and (ii) a slightly modified liquid broth assay [33] referred to as the time kill assay from 0 to 1.5 h.

For the radial diffusion assay a mid-log phase bacteria were diluted to the concentration of ~ 1–2 × 10^6^ CFU/ml in 10 ml of melted and cooled to 42°C, 1% agarose (w/v, molecular biology grade) in 10 mM sodium phosphate containing 1/100 dilution of full strength BHI and 0.02% (v/v) Tween 20 (Sigma). After vigorous mixing, the bacteria in the agarose were poured onto Petri plate forming under-layer in which 3 mm diameter sterile wells were cut. The wells were filled with serially diluted neuropeptides (5 μl) at concentrations ranging from 10^−6^ M to 10^−3^ M and plates were incubated for 3 h at 37°C to allow peptide diffusion. The plates were then overlaid with 10 ml of the buffered 1% agarose containing 6% BHI (w/v) and allowed to harden. After overnight incubation at 37°C the diameters of inhibition zones were measured and antimicrobial activities of NPs expressed as minimal inhibitory concentrations (MIC) were evaluated. The MIC value was determined as the x-axis intercept obtained from the relationship between the diameter of radial diffusion zones versus NP concentration. Resistance was defined for MIC greater than 500 μg/ml [22]. Polymyxin B was used as a positive control.

For the time kill assay from 0 to 1.5 h, overnight cultures of Mc and NTHi were inoculated into new medium and incubated under the relevant conditions until early log-phase (OD_600_ = 0.25–0.3). The bacteria were diluted in 10 mM sodium phosphate buffer, NaPB, (pH 7.4) containing 1% (w/v) BHI to obtain ~2 x 10^6^ CFU/ml. The bacterial suspensions were incubated in the presence of 10 μM and 100 μM concentrations of SP, CGRP, NPY, and SOM in a final volume of 100 μl NAPB-1% BHI, for 0, 0.5 and 1.5 h at 37°C in water bath. After each time of incubation the suspensions were 10-fold serially diluted with 10 mM NaPB-1% BHI and 20 μl aliquots were plated in triplicate on BHI (*M. catarrhalis*) or HAEM (*H. influenzae*) agar plates. The plates were incubated overnight at 37°C in 5% CO_2_ conditions. Simultaneously, the incubation of bacteria in the presence of an appropriate diluent was included for positive control of bacterial growth. The colony counts and CFU/ml were calculated next day. The bactericidal activity of neuropeptides was expressed as survival ratios determined as the numbers of CFU after neuropeptide incubation divided by the numbers of CFU estimated from a control plate (time 0). All antimicrobial assays were performed at least two times in triplicate.

### Membrane permeability assay

To measure the activity of neuropeptides in permeabilization of bacterial membrane, the ONPG-mediated β-galactosidase microplate assay was used. Permeabilization was measured using *E. coli* ML-35 with constitutive cytoplasmic β-galactosidase activity as previously described [34,35]. Briefly, 18 h *E. coli* ML-35p in TSB was recultivated in TSB until mid-log phase (OD_600_ = 0.5). Flasks were put on ice for 20 min, and 2 ml of bacterial suspensions were transferred to Eppendorf tubes, washed twice with 10 mM sodium phosphate NaPB (pH 7.4) and suspended in NaPB to OD_600_ = 0.5. Bacterial suspensions were diluted in NaPB to obtain 10^6^ CFU/ml and incubated on the flat bottomed 96-well plate (NUNC, Denmark) with various concentrations (10^−6^–10^−4^ M) of CGRP, SP, SOM, NPY, and NPY_18–36_ diluted in NaPB with 3 mM ONPG as β-galactosidase substrate in the final volume of 150 μl. Microplates were incubated at 37°C for 1.5 h and optical densities were measured every 15 minutes at λ=405 nm (spectrophotometer Opsys MR Dynex Technologist). All the assays were performed in triplicate. Two positive controls were used: polymyxin B at the final concentration of 1 μg/ml as an established model of peptide permeabilizing action, and 20% chloroform (v/v). Chloroform, as an indicator of extensive permeabilization (arbitrarily defined as maximal) which led to the maximal OD increase, was used in order to quantitatively calculate and compare the permeabilizing strength of studied NPs. We estimated their molar concentration defined as the inducible dose (ID_50_) that caused 50% increase in OD calculated with reference to the chloroform-inducible maximum, 45 min after the beginning of measurement. The molar concentration of NP that caused such increase was extrapolated from a linear portion of NP titration curve. Negative controls constituted bacteria incubated with NaPB and ONPG but without neuropeptides.

### Isolation of PMNs

Polymorphonuclear leukocytes (PMNs) from healthy young adult volunteers were isolated from venous blood samples anticoagulated with Lithium-heparin (7.5 ml) by ficoll (Aqua-Med, Poland) density gradient centrifugation (2000 rpm/30 min), followed by hypotonic lysis of erythrocytes with a lysing buffer (0.83% NH_4_Cl, 0.1% KHCO_3_, EDTA, pH 7.4). After two washes (1400 rpm/10 min) in PBS, PMNs were resuspended to the final concentration of 10^7^ cells/ml in phagocytic buffer: HBSS with Ca^++^ and Mg^++^ supplemented with 1% HiFBS. The purity of granulocyte suspensions was checked using 15 μg/ml of acridine orange. The cell suspensions containing at least 95% of PMNs were used for the phagocytosis assays described below. Cell viability was determined to be > 97% by trypan blue staining. Isolated PMNs were kept for 30 min at 37°C and 5% CO_2_ until used.

The study was approved by the Research Ethics Committee of the Medical University of Wroclaw and informed consent was obtained from all volunteers.

### Phagocytosis assay

i. Labeling of bacteria with fluorescein isothiocyanate (FITC): FITC-labeled bacteria were prepared as described previously [36]. Briefly, bacteria *M. catarrhalis* ATCC 25238 and NTHi ATCC 49247 from overnight cultures were harvested, suspended into PBS and adjusted to an *A*_600_ of 1 (OD = 1) corresponding to ~ 2 × 10^9^ CFU/ml. Heat-killed bacterial suspensions at OD = 1 were labeled with 1 mg/ml of FITC at 0.05 M carbonate/bicarbonate buffer (pH 9.5), for 30 min at 37°C with gentle mixing in the dark. FITC-conjugated bacteria were washed 3 times by centrifugation with cold carbonate/bicarbonate buffer in order to remove the excess of FITC, and finally resuspended in HBSS-Ca^++^Mg^++^ at 1/10 of original volume.

ii. Opsonization: FITC-labeled bacteria in HBSS- Ca^++^Mg^++^ were opsonized with post-immunized 10% heat-inactivated pooled murine antisera for 30 min at 37°C with rotation, followed by washing with PBS. Antisera from mice immunized with whole bacteria (Mc or NTHi) had whole cell ELISA titers of specific IgG antibodies, which were > 1: 10,000.

iii. >Bacterial uptake: 50 μl of PMNs (10^7^ cells/ml) in phagocytic buffer was mixed with 10 μl of FITC-labeled targets supplemented with 5% HiFBS and 10^−8^ M concentration of NPs in a final volume of 100 μl and incubated at 37°C for 30 min with gentle agitation on rotary shaker (100 rpm). Phagocytosis was terminated by placing the samples briefly on ice. Parallel controls were kept on ice to block endocytic uptake of the targets. Cells were treated with 0.2 mg/ml of trypan blue solution to quench extracellular fluorescence [37]. Each assay was performed at least three times in duplicate. The concentration of NPs indicated as the most potent within the range 10^−12^ to 10^−8^ M, was chosen on the basis of preliminary experiments.

iv. Flow cytometric analysis: Fluorescence of FITC-labeled bacteria ingested by granulocytes (FITC-positive granulocytes) was measured by FACS Calibur (Becton Dickinson). Before analysis the samples were diluted 1:5 in cold HBSS-Ca^++^Mg^++^. PMNs were distinguished from other leukocytes by gating on forward- and sideward-scatter signals, then this population was analyzed for green fluorescence in the FL1 channel. 15,000 events were measured to assess the percentage of phagocytizing neutrophils and the mean fluorescence intensity (MFI) of PMNs. Data were obtained using Becton Dickinson software and further analysis was performed using WinMDI 2.8 software. Data were expressed as the percentage of phagocytosis in reference to control values (100% = phagocytosis without NPs) or as the MFI of PMN.

### Detection of ROS formation by chemiluminescence

Reactive oxygen species (ROS) formation by PMNs was measured using luminol-dependent chemiluminescence assay (CL) in white flat-bottom 96-well microplates (NUNC, Denmark). The reaction mixture of total volume 200 μl in each well contained: 100 μl of PMN suspension in HBSS–Ca^++^Mg^++^ (10^5^ cells/well), 40 μl of luminol (50 μM/well), 40 μl of fMLP (0.2 μM/well), 20 μl of particular NP (in the range 10^−12^–10^−8^ M/well). In other experiments, the respiratory burst was triggered by phagocytosis of 40 μl heat killed (56°C/30 min) *M. catarrhalis* ATCC 25238 or *H. influenzae* ATCC 49247 at concentration of ½ of OD_600_ = 1 or OD_600_ = 1, respectively. In these experiments, only the most potent NPs concentration (10^−8^ M) was used. The PMA stimulus at 0.1 μM final concentration was used as positive control of the relevant oxidative stress. The CL kinetics was measured at constant 37°C temperature using luminometer (Microlumat, LB96P, Berthold) and recorded as relative light units (RLU). Measuring conditions for fMLP were: interval (46 s), run time (up to 30 min). The conditions for bacterial and PMA stimulation were: interval (83 s), run time (up to 60 min). The maximal value of CL response curve was expressed as peak height (PH_CL_) for fMLP stimulant. In other cases the area under chemiluminescence curve (AUC_CL_) was calculated [38]. Each sample was run in triplicate and the values were averaged. Relative CL was calculated as: mean AUC_CL of sample_ / mean AUC_CL of control_ × 100%. Results were expressed as mean ± SD. After measurements the viability of PMNs was confirmed to be > 93% using trypan blue exclusion.

### Relationship between the direct antibacterial activity of peptides and their physicochemical characteristics

The relationship of neuropepeptide activities with their different physicochemical properties and amino acid composition were correlated using Spearman’s rank coefficient. The antibacterial properties of the analyzed neuropeptides were expressed as survival ratios after 1.5 h of bacterial exposure whereas permeability properties were measured by peptide concentration (expressed in μM) that caused 50% increase (ID_50_) in the membrane permeability (measured by OD) after 45 minutes from the beginning of the experiment. In the analyses we also included the antibacterial properties (expressed as MIC in μM) of neuropeptides/peptides published by other scientists [21,23]. The data from experiments performed on different bacteria but for the same or overlapping set of peptides were min-max normalized to be included in one meta-analysis. Ten physicochemical properties deduced from sequences of the peptides were considered. Isoelectric point was calculated based on the standard iterative algorithm used in ExPASy Proteomics Server [39]. Appropriate amino acid indices downloaded from ExPASy Proteomics Server were applied to calculate polarity [40], hydropathicity [41], transmembrane tendency [42], and conformational parameter for α-helix and β-sheet [43]. Maximum hydrophobic moment was calculated as implemented in hmoment from EMBOSS package [44]. The NPs composition of amino acids classified to four different physicochemical groups: non-polar (A, C, F, G, I, L, M, P, V, W), polar uncharged (N, Q, S, T, Y), basic (H, K, R), and acidic (D, E) was analyzed.

### Statistical analysis

Normality and homogeneity of variance assumptions were checked using Shapiro-Wilk’s and Levenea’s test, respectively. For multiple comparisons, data were analyzed using one-factor analyses of variance (ANOVA) and were followed by post hoc effects (Tuckey’s test or Duncan’s test). Spearman’s rank correlation coefficients were used for the relationship assessment. Results were expressed as means ± SEM of the *n* independent experiments performed in triplicate and containing cells from separate donors. For the comparison of ROS generation or phagocytic uptake by bacteria-stimulated PMNs in response to neuropeptides, the data were normalized to a baseline control value (without NPs) of 100%. A *P* value < 0.05 was used to assess the significance of all statistical analyses. All the analyses were performed with STATISTICA 9 software.

## Results

### Rank order of permeabilizing potency of neuropeptides

In order to determine the potency of CGRP, NPY, its truncated fragment NPY_18–36_, SOM and SP on permeability of the gram-negative bacteria membrane, a model microorganism, *E. coli* ML-35p and β-galactosidase assay were used. The time-dependent increase in absorbance at 405 nm in this assay is the result of permeability of both the inner and outer bacterial membranes, confirmed by the intracellular influx of the extracellular dye ONPG that is hydrolyzed by constitutively expressed β-galactosidase [34]. In each set of experiments, polymyxin B was used as the first positive control and the established model of peptide permeabilizing action. The second positive control employed chloroform which caused an extensive bacterial membrane permeabilization, and led to the maximal OD increase.

**Figure 1:**
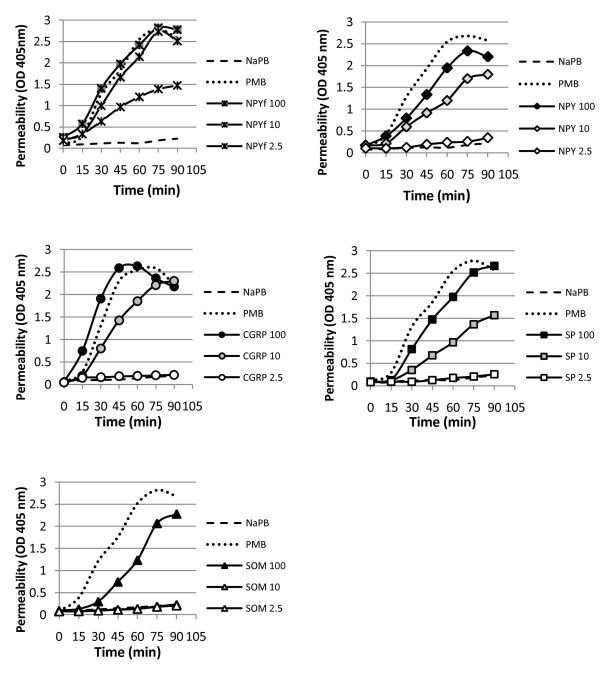
**Permeabilizing potency of neuropeptides against*E.coli*ML-35p, determined by β-galactosidase assay with ONPG as its substrate.** Bacteria at 10^6^ CFU/ml were incubated at 37°C for 90 min in the presence of the indicated micromolar concentrations of neuropeptides: neuropeptide Y (NPY), fragment of neuropeptide Y (NPYf = NPY_18–36_), calcitonin gene related peptide (CGRP), substance P (SP), and somatostatin (SOM). In negative controls, bacteria incubated in neuropeptide-free NAPB containing 1/100 of TSB showed no net hydrolysis of ONPG. Polymyxin B (PMB) at 1 μg/ml was included as a positive control. The representative curves of at least three independent experiments are presented.

Tested neuropeptides displayed an activity in bacterial membrane permeability in a time- and dose-dependent manner (Figure 1). An examination of the neuropeptide-induced ONPG influx, as a function of time indicates that the shapes of permeability profiles were similar whereas time courses of the maximal permeability for all tested NPs used at highest (100 μM) concentrations were slightly different. CGRP induced the maximal OD increase at 50 min, NPY and NPY_18–36_ at 75 min, whereas SP and SOM at 90 min from the beginning of the reaction, followed by a gradual decrease in OD over time (not shown). Concentrations of 10 μM exerted permeabilizing potency for all tested neuropeptides except SOM, and induced a maximal OD increase at ~ 90 min of ONPG influx (only for NPY_18–36_ it was ~ 75 min). To quantitatively calculate the permeabilizing potency of NPs, we estimated their molar concentration defined as the inducible dose (ID_50_) that caused 50% increase in OD calculated in reference to the chloroform-inducible maximum at 45 min. The molar concentration of NPs calculated for different times and raw permeability values led to the same final conclusion. The neuropeptide concentrations needed for 50% ONPG influx are presented in Table 1. Their permeability potency against *E. coli* ML-35p increased in the following order: SOM < < SP < NPY ≤ CGRP < NPY_18–36_. These findings demonstrate that the four studied NPs efficiently permeabilized bacterial membranes of the model microorganism *E. coli* ML-35p with NPY_18–36_ being the most effective and SOM the least.

**Table 1:** **Parameter defining NP-induced intracellular influx of ONPG into*E.coli*ML-35p**

Neuropeptide	*ID_50_ [μM]
NPY_18–36_	2.0
NPY	19.3
CGRP	11.5
SP	29.2
SOM	104.1

*ID_50_ (inducible dose) - dose of NP that caused 50% increase in OD calculated in the reference to the chloroform-inducible maximum at 45 min of experiment. The values carry a standard error of approximately.

### Killing efficacy of *M. catarrhalis* and *H. influenzae* by CGRP, NPY, SP, and SOM

The permeabilizing potency of peptide may sometimes not correlate with its lethal effect. Moreover, there are some discrepancies concerning the direct antibacterial action of neuropeptides used in this study [22,23], and their activity against clinical respiratory pathogens has not been elucidated so far. Thus in the next step we compared the killing efficacy of effective permeabilizing neuropeptide doses (10 and 10^2^ μM) against isolates of *M. catarrhalis* and *H. influenzae* using two methods: (i) a radial diffusion assay and (ii) a time kill assay from 0 to 1.5 h. In the radial diffusion assay CGRP, NPY, NPY_18–36_, SP, and SOM were tested against *M. catarrhalis* ATCC 25238, *H. influenzae* ATCC 49247, and four clinical respiratory isolates of *M. catarrhalis* (Mc5, Mc6) and non-typeable *H. influenzae* (NTHi3, NTHi6). In this assay, within used NPs ranges from 1 μM to 10^2^ μM, all tested *M. catarrhalis* and *H. influenzae* strains were susceptible only to the truncated form of neuropeptide Y (NPY_18–36_). *M. catarrhalis* strains were generally two/three-fold more susceptible (MICs from 8 to 12.5 μM) than *H. influenzae* (MICs from 25 to 40 μM). In order to test if higher doses of NPs (apart from NPY_18–36_) can be bactericidal, two additional concentrations, 5 x 10^2^ μM and 10^3^ μM were used. Weak inhibition zones were observed for four NPs at indicated concentrations. CGRP, NPY, SP, and SOM exhibited no antimicrobial activity in the radial diffusion assay as their MICs for all 6 isolates of both *M. catarrhalis* and *H. influenzae* were higher than 117 μM (NPY), 132 μM (CGRP) and 300 μM (SP and SOM) which corresponded to 500 μg/ml, defined as the *cut off* for antimicrobial neuropeptide activity [22,23]. The similar results were obtained for additional tested isolates of both species (data not shown). In control experiments all tested *M. catarrhalis* and *H. influenza*e isolates showed susceptibility to polymyxin B within a range from 0.39 μg/ml to 3.12 μg/ml and from 1.56 μg/ml to 6.25 μg/ml, respectively.

**Figure 2:**
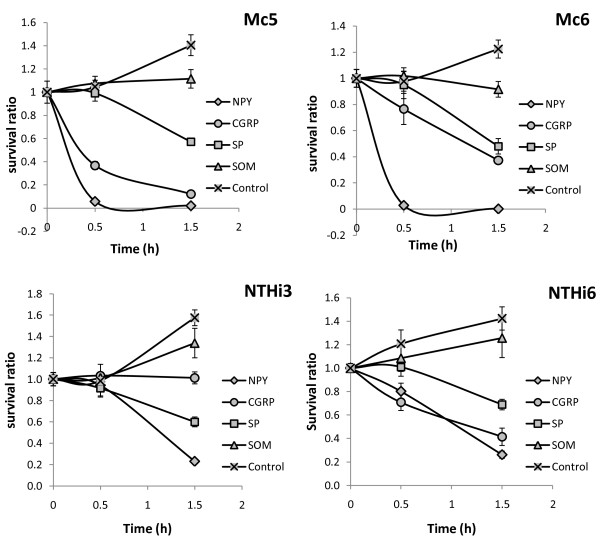
**Neuropeptide-mediated killing of*M. catarrhalis*(Mc) and nontypeable*H. influenzae*(NTHi).** Bacteria at ~ 2 × 10^6^ CFU/ml were incubated at NaPB - 1% BHI (v/w) containing 100 μM concentration of NPY, CGRP, SP, and SOM for 0.5 and 1.5 h. Serially diluted aliquotes were plated on BHI agar. The data are expressed as mean survival ratios ± SEM from two independent experiments performed in triplicate.

In contrast to the radial diffusion assay, three studied neuropeptides (except SOM) exerted killing effect in the time kill assay from 0 to 1.5 h. As illustrated on survival plots (Figure 2). *M. catarrhalis* strains were in general more susceptible to the tested neuropeptides than *H. influenzae*. Use of 100 μM concentration of CGRP, NPY, and SP against *M. catarrhalis* resulted in killing of both tested Mc strains that occurred in a time-dependent manner. NPY killed more than 95% of bacterial cells of both strains after 0.5 h and nearly 99% of them after 1.5 h. CGRP was less potent than NPY in killing *M. catarrhalis*. In its presence ~40% of Mc5 and more than 20% of Mc6 cells were killed after 0.5 h incubation, and respectively nearly 90% and more than 60% were killed after 1.5 h. The lethal activity of SP against Mc5 and Mc6 strains was similar. SP was bactericidal for~ 43% of Mc5 cells and 52% of Mc6 cells after 1.5 h, whereas after 0.5 h incubation nearly 100% of both cultures survived. Interestingly 100 μM SOM elicited bacteriostatic activity for both Mc strains. There was no *M. catarrhalis* growth inhibition when all NPs were used at 10 μM (data not shown). All neuropeptides displayed a similar pattern of killing activity against *H. influenzae* strains NTHi3 and NTHi6. Similarly to *M. catarrhalis*, no growth inhibition was observed for any NPs used at a 10 μM concentration. The evaluation of NTHi survival revealed that in the presence of the most potent 100 μM NPY ~75% of NTHi3 and NTHi6 were killed after 1.5 h incubation whereas after 0.5 h NPY led to the death of up to 20% of the bacteria. 100 μM SP was able to reduce the bacterial viability of both strains by at least 30% after 1.5 h. CGRP killed ~ 30% of NTHi6 after 0.5 h and ~ 60% after 1.5 h, whereas for NTHi3 its effect was bacteriostatic. The incubation of Mc and NTHi strains without neuropeptides (controls) showed an approximate 1.5-fold increase of bacterial growth after 1.5 h of incubation. Extension of incubation of Mc and NTHi strains in the presence of NPs up to the 3 h resulted in the abolishment of NP-mediated bactericidal activity and in a gradual increase in bacterial growth (data not shown), indicating that NPs are efficient in killing mucosal pathogens within a short time period.

Thus, apart from bacteriostatic SOM, 100 μM concentration of CGRP, NPY, and SP are bactericidal against clinical isolates of *M. catarrhalis* and *H. influenza*e. NP-mediated killing occurred within 1.5 hours of exposure in a time-dependent manner. These data also indicate that the rank order of bactericidal activity of 100 μM NPs was similar to that of the permeabilizing potency with NPY > CGRP ≥ SP >> SOM. Overall, these data indicate that CGRP, NPY, SP and SOM at micromolar concentrations have the capacity, respectively to kill high numbers of mucosal pathogens and inhibit their growth within short time period.

### Relationships between the direct antibacterial activities of peptides and their physicochemical characteristics

**Figure 3:**
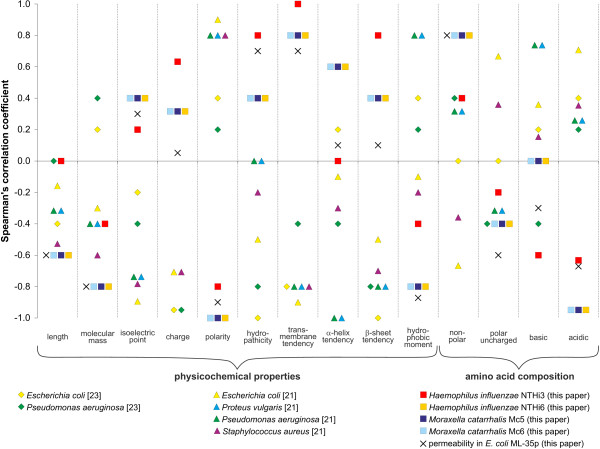
**Spearman’s rank correlation coefficient between antibacterial activity of peptides and their physicochemical characterization.** An individual symbol represents the result of a given experiment. Experiments presented in this paper involved: calcitonin gene-related peptide, neuropeptide Y, fragment of neuropeptide Y (NPYf = NPY_18–36_), substance P, and somatostatin. Data taken from Kowalska et al. [21] included: bradykinin, indolicidin, neurotensin, substance P, and substance P antagonist whereas data from El Karim et al. [23] contained: calcitonin gene-related peptide, neuropeptide Y, substance P, and vasoactive intestinal peptide. For further explanation see Materials and Methods.

Some relationships between antimicrobial effect of the endogenous peptides and their amino acid composition or physicochemical properties have been reported [45]. Most of these peptides form cationic amphipathic secondary structures, typically α-helices and β-sheets with high hydrophobicity. In order to assess these relationships quantitatively for the analyzed neuropeptides, we correlated their permeability effectiveness and killing efficacy expressed as survival ratios with their different physicochemical properties and amino acid composition. We also compared the results with data for other peptide sets with experimentally determined antimicrobial activity gathered from papers [21,23]. Spearman’s rank correlation coefficients between antibacterial activity of peptides and their physicochemical characterization for particular experiments were presented in Figure 3, whereas coefficients shown in Table 2 were calculated for min-max normalized data from several experiments performed on different bacteria and for the same or overlapping set of peptides. 

**Table 2:** Spearman’s rank correlation coefficient (with p-value) between antibacterial activity of peptides and their physicochemical characterization including data from experiments carried out in this paper and by other authors

Physicochemical characteristics	Peptide set analysed in:		
	this paper	[21]	[23]
length	**−0.44 (0.044)**	−0.33 (0.174)	−0.25 (0.555)
molecular mass	**−0.69 (0.000)**	−0.42 (0.085)	0.27 (0.515)
isoelectric point	0.31 (0.164)	**−0.78 (0.000)**	−0.27 (0.515)
charge	0.32 (0.160)	**−0.48 (0.043)**	**−0.94 (0.001)**
polarity	**−0.91 (0.000)**	**0.79 (0.000)**	0.32 (0.438)
hydropathicity	**0.57 (0.006)**	−0.14 (0.572)	**−0.91 (0.002)**
transmembrane tendency	**0.82 (0.000)**	**−0.79 (0.000)**	−0.64 (0.086)
α-helix tendency	0.31 (0.170)	**−0.58 (0.012)**	−0.02 (0.954)
β-sheet tendency	0.41 (0.063)	**−0.66 (0.003)**	**−0.91 (0.002)**
maximum hydrophobic moment	**−0.72 (0.000)**	0.26 (0.306)	0.32 (0.438)
non-polar residues [%]	**0.70 (0.000)**	−0.13 (0.600)	0.15 (0.726)
polar residues [%]	−0.41 (0.063)	0.13 (0.600)	−0.15 (0.726)
basic residues [%]	−0.23 (0.316)	0.44 (0.065)	−0.02 (0.954)
acidic residues [%]	**−0.79 (0.000)**	0.38 (0.123)	0.32 (0.438)

The data from experiments performed on different bacteria but for the same or overlapping set of peptides’ set were min-max normalized and included in one analysis. For further explanation see Materials and Methods. Results significant with *p*-value < 0.05 were shown in bold.

The correlations obtained for the peptides studied in this paper were very similar, irrespective of the antimicrobial measure: effectiveness of permeabilization or bacterial killing (Figure 3). The antimicrobial activity increased significantly with length, molecular mass, polarity, hydrophobic moment, and content of acidic residues in the peptides whereas it decreased with their tendency to form transmembrane domains, content of non-polar amino acid residues and hydropathicity (Table 1). However, the antibacterial potency for the sets of peptides taken from [21] increased significantly with isoelectric point, charge, tendency to form transmembrane domains and α-helices or β-sheet structures whereas decreased with polarity (Table 1). According to data by [23], peptides with larger charge, hydropathicity and tendency to form β-sheet structures showed higher activity against bacteria (Table 1).

### NP-mediated modulation of bacterial uptake by PMNs

**Figure 4:**
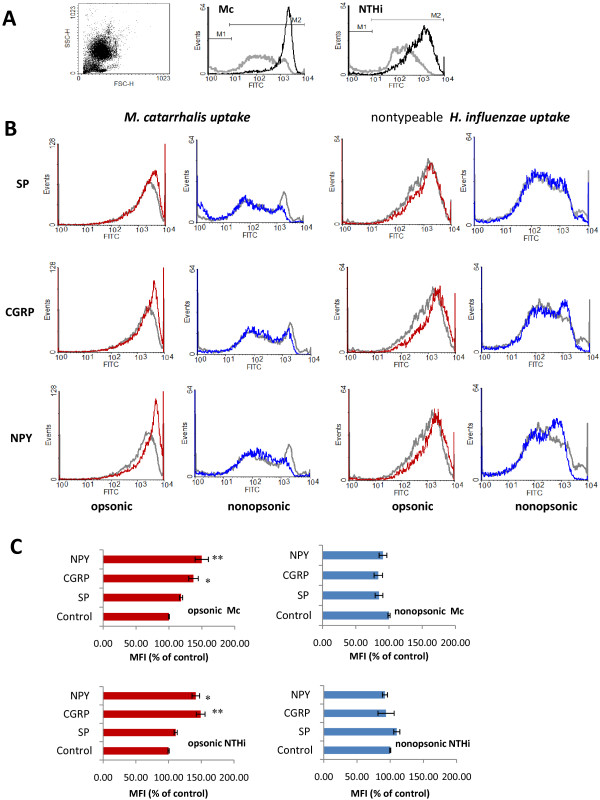
**Involvement of various neuropeptides (NPs) used at 10**^**−8**^ **M in the modulation of heat-inactivated serum opsonized and nonopsonized*M. catarrhalis*ATCC 25238 (Mc) and*H. influenzae*ATCC 49247 (NTHi) engulfment by isolated human polymorphonuclear phagocytes (PMNs) analyzed by flow cytometry.** PMNs were incubated with FITC-labeled bacteria for 30 min at 37°C with agitation and analyses of 15,000 PMNs are presented. Shown is fluorescence signal indicated as geometric mean of the FL-1 signal from the gated sample with phagocytosing PMNs*.***(A)** Representative FACs dot plot with gated PMNs and representative histograms of phagocytic uptake of serum-opsonized (black lines) and nonopsonized (grey lines) bacteria by PMNs in the absence of NPs are shown. **(B)** Histograms represent increased or decreased endocytic uptake of FITC-labeled opsonized versus nonopsonized targets in the presence of indicated neuropeptides (10^−8^ M). The levels of bacteria ingested, indicated by mean fluorescence intensity (MFI) values are shown. Color lines show test samples in the presence of indicated neuropeptides, grey lines represent bacterial uptake in the absence of neuropeptides. Results are from one experiment representative of three experiments done with separate blood donors. **(C)** Data are expressed as the percentage of phagocytosis reported with respect to control values (100% = phagocytosis without NPs). Values are means ± SEM of at least n = 5 (*M. catarrhalis*) and n = 3 (*H. influenzae*) independent experiments. Differences between control (without NPs) and treatment condition uptake are assigned by asterisks and considered significant if ^*^p < 0.005 and ^**^p < 0.0005 (one-way ANOVA followed by Tuckey’s post hock test).

NP-induced phagocytosis may be triggered as a result of both non-specific and specific ligand-receptor interaction and may depend on the nature of the engulfed pathogen [8,46]. We investigated whether SP, NPY, and CGRP exert a modulating role in the uptake of mucosal pathogens by freshly isolated human granulocytes, and whether such modulation involves a distinct type of pathogen recognition (opsonic versus nonopsonic phagocytosis). As expected, precoating *M. catarrhalis* with 10% antiserum significantly enhanced the phagocytic capacity of PMNs (MFI 1411.2 ± 111.8 (SEM); n = 6) in comparison to nonopsonic conditions (MFI 289.7 ± 51.5; n = 6; *p* < 0.004) and respectively nearly 97% ± 0.49% versus 83% ± 3.9% of FITC-positive granulocytes (n = 6; *p* < 0.02) were detected by FACs. Uptake of *H. influenzae* revealed a similar 4-fold increase under opsonic versus non-opsonic conditions (Figure 4A). The observed opsonic phagocytic potency of PMNs against both mucosal pathogens was next markedly improved in the presence of NPs (Figure 4B). As shown in Figure 4C the enhancement of serum opsonized *M. catarrhalis* or *H. influenzae* uptake was from ~18% to ~ 50% and from ~ 11% to nearly 42%, respectively, depending on the tested NP with evident potency of NPY ≥ CGRP > SP. These results suggest that the stimulatory effects of NPY, CGRP, and SP on PMN uptake seem to be additive to the opsonin action. Figure 4B and C demonstrate that, when nonopsonized *M. catarrhalis* or *H. influenzae* cells were ingested by PMNs, their uptake in the presence of neuropeptides remained unchanged or was slightly diminished (up to 15% inhibition) compared to non-stimulated positive control indicating no effect or slightly inhibitory action. Moreover, as expected, the differences in magnitude of the modulating properties of NPs were also dependent on the PMN source thereby reflecting the actual physiologic conditions and vital responsiveness of PMNs from various donors. We postulate from this finding a stimulating action of the NPs, which depends on the phagocytic recognition of the studied mucosal pathogens and is connected with opsonin presence. 

### NP-mediated modulation of ROS metabolism by PMNs

**Figure 5:**
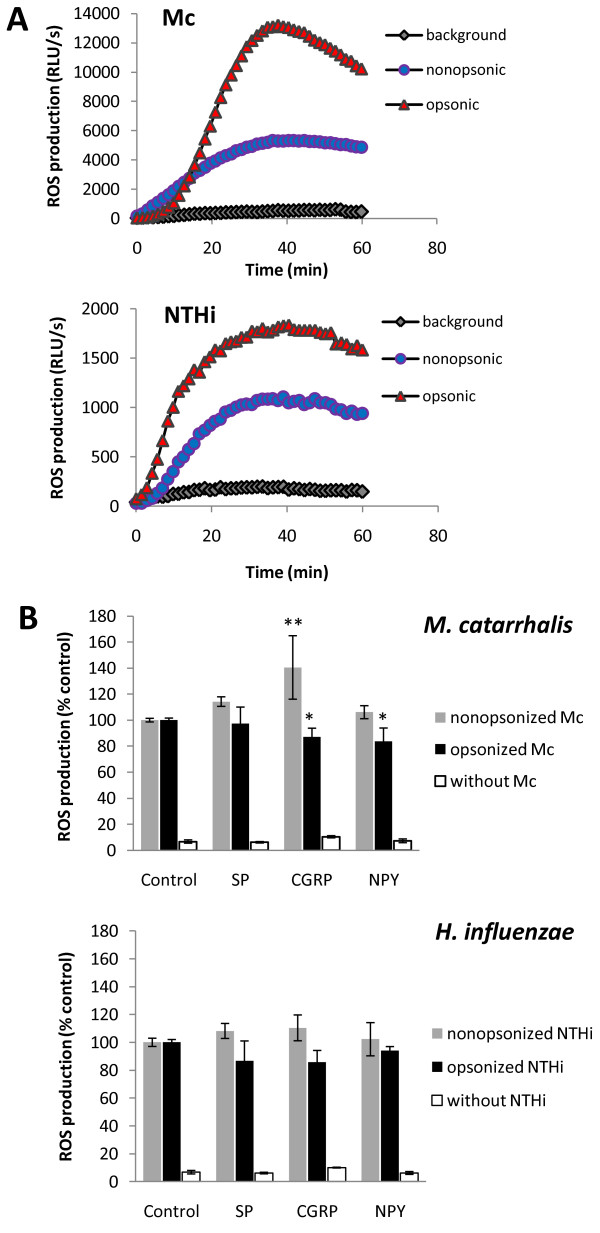
**Influence of neuropeptides on ROS generation by human PMNs.** Human PMNs (10^5^/well) were added to ~10^7^ CFU of nonopsonized or 10% heat-inactivated serum-opsonized *M. catarrhalis* ATCC 25238 (Mc) or nontypeable *H. influenzae* ATCC 49247 (NTHi) or to wells without bacteria in the presence or absence of 10^−8^ M of given neuropeptides and luminol-dependent chemiluminescence was measured up to 60 min using Berthold Microlumat luminometer. **(A)** Kinetics of ROS generation in human PMNs in response to bacterial stimulation in the absence of NPs. The results are expressed as mean relative light units/sec (RLU/s). The representative experiment performed in triplicate is shown **(B)** Histograms represent the AUC_CL_ (mean ± SD) of at least 3 independent experiments done in triplicate from different donors. Results are expressed as a percentage of the PMN response stimulated only with bacteria. Asterisks indicate statistically significant differences calculated with reference to relevant controls if ^*^p ≤ 0.05 and ^**^p < 0.0005 (one-way ANOVA followed by Duncan’s post hock test).

The luminol-dependent chemiluminescence (CL) response was used to measure the bactericidal activity of PMNs. Since the kinetics of the CL response triggered by fMLF and phagocytosable bacteria or PMA, including both the magnitude and the temporal pattern were quite different, we used in calculations PH_CL_ and AUC_CL_, respectively. In preliminary experiments, in order to choose the most effective modulating doses of neuropeptides, fMLF stimulation was used. This stimulation generated a sharp maximum, which was observed within ~5 min of ROS response, followed by a subsequent decline to the baseline. The significant stimulatory effects of various physiological doses of neuropeptides (10^−12^ M–10^−8^ M) on the luminol-dependent CL responses of PMNs activated by fMLF are shown in Table 3. The amplification of ROS release, with maximal effect observed at ~5 min of fMLF exposure, was more potent under lower NP concentrations with the rank order of CGRP > SP > NPY. Neuropeptides CGRP, SP, and NPY alone did not activate a respiratory burst of PMNs. To evaluate whether the NP-mediated intensification of respiratory burst by PMNs was more relevant to PMN physiology, the respiratory burst activity was determined following stimulation by *M. catarrhalis* ATCC 25238 and NTHI ATCC 49247. Additionally, we explored the question if such modulation involves distinct types of pathogen recognition (opsonic versus nonopsonic). Incubation of human PMNs with opsonized as well as nonopsonized *M. catarrhalis* or nontypeable *H. influenzae* resulted in substantial ROS generation, peaking between 35 and 40 min, followed by a gradual decrease. As shown in Figure 5A, for a representative experiment, the opsonization with 10% heat inactivated antiserum increased both the rate and the total production of ROS, which for both studied bacteria, was approximately 2-fold higher comparing to nonopsonic stimulation. In this case, log_10_ of mean AUC_CL_ ± SEM for opsonized versus nonopsonized *M. catarrhalis* and *H. influenzae* was 5.71 ± 4.67 versus 5.39 ± 4.47 and 4.99 ± 3.62 versus 4.69 ± 3.3, respectively. As shown in Figure 5B, only CGRP used at 10^−8^ M significantly intensified the respiratory burst of PMNs triggered by phagocytosable *M. catarrhalis* in the absence of serum, thus confirming partly the results obtained with soluble fMLF. There were, however, some differences in the sensitivity of the PMNs responses to the individual bacterial stimulant as well as interindividual variation in ROS generation. ROS production by PMNs to *M. catarrhalis* was generally more sensitive to NPs than equivalent response to NTHi. Data obtained for *M. catarrhalis* (Figure 5B) show that the mean AUC_CL_ of PMNs stimulated with these bacteria was increased in the presence of SP by 14.2 ± 3.4% (range: 10.3−20.1%, n = 6), CGRP by 40.5 ± 22.3% (range: 22.7−75.7%, n = 6) and NPY by 7.5 ± 4.0% (range: 3.5−12.8%, n = 5). The stimulatory effect of NPs in the presence of NTHi was relatively weaker. The mean relative CL increase elicited (4 donors) was: SP 8.2 ± 5.4 (range: 3.2−17.0%), CGRP 10.4 ± 9.3 (range: 0.0−25.0%) and NPY 2.2 ± 9.3 (range: 0.0−12.7%). The observed stimulating effect of NPs was not preserved for both opsonized bacteria (Figure 5B). Under these conditions the relative amounts of ROS were usually diminished up to 15% below the level of control ROS production. It indicated an inhibitory rather than stimulatory action of CGRP and inhibitory action of NPY during the response to *M. catarrhalis*. The similar modulating tendency of NPs, although not significant, was shown for NTHi. PMNs without bacteria or those stimulated only with NPs did not release significant amounts of ROS. The analysis of PMA-induced CL in PMNs was included in each experiment as an internal standard of relevant PMN activation. A stimulation with PMA always generated the strongest and slightly biphasic response with usually two separate maxima (~10 min, and ~25 min) which gradually decreased. The log_10_ of mean AUC_CL_ ± SEM calculated from 0 to 60 min for that soluble and stabile stimulant was 6.05 ± 4.32 (not shown data from five independent experiments done in triplicate, using PMNs from 5 blood donors). Taken together, the presented data suggest that CGRP and less NPY at physiological concentrations are modulators of the respiratory burst of PMNs in response to *M. catarrhalis*, whereas in the case of nontypeable *H. influenzae* these effects are notable for CGRP and SP.

**Table 3:** Neuropeptide-mediated stimulation of fMLF-induced ROS generation by PMNs in luminol-dependent chemiluminescence

Stimulus	ROS release [% of control]								
	SP			NPY			CGRP		
	10^-8^ M	10^-10^ M	10^-12^ M	10^-8^ M	10^-10^ M	10^-12^ M	10^-8^ M	10^-10^ M	10^-12^ M
fMLF	179.18^a^	160.36^a^	140.83^a^	113.33	136.04^a^	129.67^c^	266.41^a^	164.35^a^	153.47^b^
	(4.99)	(3.80)	(5.22)	(6.64)	(3.91)	(3.26)	(8.37)	(13.47)	(1.5)

Human PMNs (10^5^ cells/well) were incubated in the presence of 0.2 μM of fMLF and three concentrations of each NPs and chemiluminescence was measured up to 30 min using Berthold Microlumat luminometer. Values are expressed as relative changes from controls (PMNs + fMLF) and were calculated based on the maximal CL increase (PH_CL_). The presented results are means ± (SEM) of three independent experiments performed at least in triplicate. ^a-c^ Significantly different from fMLF-stimulated ROS increase (^a^*p* < 0.0005; ^b^*p* < 0.001; ^c^*p* < 0.05) as determined by Tuckey’s post hock test.

## Discussion

*Moraxella catarrhalis* and nontypeable *Haemophilus influenzae* are human-restricted mucosal pathogens that may potentially be exposed to the action of locally released neuropeptides. The importance of neuropeptides as the initial innate defenders seems to be associated with their direct (bactericidal) and indirect (modulatory) activity. The findings of the study demonstrate the complex antibacterial activity of neuropeptides CGRP, NPY, SP, and partially also SOM, against *M. catarrhalis* and nontypeable *H. influenzae*. Initially, we evaluated the direct antibacterial activity of neuropeptides, and found, using model *E. coli* ML-35p strain, that CGRP, NPY, NPY_18−36_ and SP at concentrations of 100 μM and 10 μM, and SOM at 100 μM are effective permeabilizers. The activity of NPs was time and dose-dependent with the following potency order of permeabilization: NPY_18−36_ > CGRP ≥ NPY > SP >>SOM. This effect was probably the result of neuropeptide-mediated sequential permeabilization of the outer and inner membrane of gram-negative bacteria since such mechanism is dedicated to other endogenous antimicrobial peptides [34,47].

The permeabilizing activity of peptides sometimes may not correlate with their killing properties [48]. Thus, our next experiments aimed at determining the nature of antibacterial activity of effective permeabilizing NP doses, more specifically, whether they are bactericidal or bacteriostatic? We found that 100 μM CGRP, NPY, and SP exerted bactericidal and time-dependent impact on the metabolically active *M. catarrhalis* and *H. influenzae* within 1.5 h. This indicated, at micromolar concentrations, their temporary capacity to kill high numbers of respiratory pathogens. The action of SOM proved, at the same time, to be bacteriostatic. The following potency order of bactericidal action was established: NPY > CGRP ≥ SP >> SOM. In general, neuropeptides start to kill *M. catarrhalis* within 0.5 h, whereas the killing of *H. influenzae* is slightly delayed in time. Threshold concentration of NPs being similar, this would indicate that the kinetics of the lethal effect depends on individual peptide-membrane interactions.

Amounts of NPs regarded as physiological (10^−12^ to 10^−8^ M) imply that bactericidal activities of NPs defined in this study are of only pharmaceutical significance. Nevertheless, we cannot rule out that neuropeptide levels may be locally elevated in response to infection or other inflammatory condition. An example of such neuropeptide alteration may be the marked increase of SP expression in the airways, following respiratory syncytial virus infection [49]. Additionally, despite the fact that neuropeptides are released from nerve endings, they can also be synthesized in and released from the immune cells such as lymphocytes, monocytes/macrophages [17-19] as well as airway epithelial cells [50] or fibroblasts [51]. Therefore, locally high concentration of NPs, sufficient for bactericidal activity, is possible under certain inflammatory state in the respiratory tract.

Ten-fold lower concentration (10 μM) of all NPs was not effective. Nevertheless, despite the lack of lethal activity, SOM being an exception, it altered the permeability barrier of model *E. coli* ML-35 mutant. Hypothetically, such an alteration could promote an interaction with final intracellular targets for a variety of extracellular molecules, including the neuropeptide itself [47]. In that case, the potential neuropeptide-mediated induction of the bacterial sensitization to other endogenic (defensins) or egzogenic (antibiotics) compounds could indirectly contribute to the local antimicrobial activity of the host or to the antimicrobial activity enhanced by chemiotherapeutics. The alliance between the cationic peptide magainin II and the betalactam antibiotics against *Pseudomonas aeruginosa*[52] may be an example of such synergism. However, the potential role of synergistic reactions between neuropeptides and others antimicrobials remains to be determined.

The direct antibacterial potential of substance P against laboratory strains of *E. coliP. aeruginosaP. vulgarisE. faecalis,* and *S. aureus* was firstly reported in 2002 using the microdilution broth method [21]. Conversely, in 2006, using radial diffusion assay, it was demonstrated a lack of the antibacterial effect of substance P and neuropeptide Y on laboratory strains of *S. aureus* and *P. aeruginosa*[22]. Recently, antimicrobial activities of various neuropeptides against a wide range of laboratory microorganisms (*S. mutansL. acidophilusE. coliE. faecalisP. aeruginosaC. albicans*) typical for skin, oral cavity, respiratory tract and gastrointestinal tract has been reported [23]. These discrepancies may be the result of different susceptibilities of strains used in the above studies, as many human cationic peptides demonstrate a strain-selective activity against microorganisms [53]. Another reason may be the different detection limits of particular methods employed. For example, a radial diffusion assay showed the resistance of *Porphyromonas gingivalis* ATCC 49417 strain to β-defensins [53], whereas a microtiter method and a DNA binding dye demonstrated its sensitivity to the same antimicrobials [54]. It has been demonstrated that subtle changes in methods for testing cationic peptides bring about marked differences in their activity [48]. Our results seem to confirm that explanation, since, unlike the liquid broth time kill assay in which 100 μM CGRP, NPY, and SP displayed killing activity against ~2 × 10^6^ CFU/ml of selected clinical isolates, such activity against the same bacterial load was not observed, when using a radial diffusion assay.

Using the data for the peptides analyzed in this paper and by other authors [21,23] we assessed the relationships between direct antibacterial activities of these peptides and their physicochemical properties as well as the amino acid composition deduced from their sequences. The performed meta-analyses revealed statistically significant relationships between many analyzed parameters. These relationships did not always show consistent trends for all considered experiments and seemed to depend rather on the tested set of peptides than the studied bacterial species or strains. The non-universality of these tendencies and its dependence on peptide sets may indicate that a similar antimicrobial activity can be achieved by peptides with different characteristics or that variable degree of this activity is exerted by the same peptide due to various experimental conditions applied for different bacterial species tested (e.g. properties of their envelopes). It is also likely that the antibacterial activity of peptides results from a superposition of many physicochemical properties, whose contribution to and influence on this activity may be different in various antimicrobial peptides that fold into different structures. Therefore, some observed specificities of NPs may result from their potential ability to form other bonds which are specific in each particular peptide-membrane system. These bonds, such as for example hydrogen bonds, are likely to cause specific configurational and conformational modification of lipid bilayer and the membrane proteins, respectively. All mentioned above consequences of the bonds may change the antibacterial peptide capacity. Our observations confirm that peptide-membrane interactions are determined by a sensitive balance of electrostatic and hydrophobic interactions in which the universal, simple correlation between antimicrobial activity and specified structural feature of peptide is seldom demonstrated [45]. More extensive studies on the antimicrobial potency of many peptides under the same experimental conditions are necessary to evaluate more precisely the relationships between their activities and physicochemical features.

Polymorphonuclear neutrophils are the first leukocyte population that comes to the site of inflammation and play a key role in the eradication of invading pathogens. The next focus of our study was therefore on the immunomodulatory effects of NPs on important steps of granulocyte phagocytosis. We addressed the question whether granulocyte-mediated uptake and the generation of oxygen radicals during phagocytosis of *M. catarrhalis* and *H. influenzae* was modulated by NPY, SP, CGRP and if such modulation involved distinct types of pathogen recognition. As expected, precoating with serum immunoglobulins significantly enhanced the phagocytic uptake by PMNs and the ability of bacterial stimuli to release ROS from human PMNs. Next, we found that all the studied NPs accelerate the phagocytic uptake of both mucosal pathogens but only in the presence of specific opsonins, whereas the stimulatory effect of NPs was not observed in their absence. Thus, the effect of NPs seems to be additive with that produced by opsonized bacteria.

Unlike phagocytic uptake, the results of the NPs impact on bacteria-stimulated ROS response showed quite different modulatory effect. We reported that NPs stimulated the respiratory burst of PMNs in the absence of opsonins while reducing oxidative burst in response to serum-treated bacteria. Under the conditions of our assays, this effect appeared to be not restricted to *M. catarrhalis*-induced response, since response to NTHi was also diminished. The finding that NPs increase the uptake of opsonized bacteria without the intensifying the respiratory burst suggests that NPs may promote bacterial uptake without exacerbating oxidant release. It follows therefore that the NP-mediated amplification of bacteria-stimulated redox metabolism of PMNs, which is required for the generation of bactericidal oxygenating agents, may be attributed only to the initial phagocytic response against the studied bacteria, that do not involve antibody cooperation. In fact, cationic peptides, found to be critical for the interaction with endotoxin, could directly enhance ROS amplification by pathogen opsonization or by exerting synergistic effect together with endotoxin [55,56]. Additionally, the insensitivity of the opsonin-dependent ROS response to NPs may indicate that its origin is probably not connected to Ig-dependent signaling. These findings strengthen the observation of bimodal action of NPY [8] and probably other NPs. Furthermore, they suggest that depending on the different receptor interplay, an efficient pathogen uptake and their effective elimination may be provided. These results also suggest that the increase in NP-mediated bacterial uptake and the ROS formation in the response to bacterial stimuli are separate and distinguishable characteristics of PMNs.

Although demonstrating the modulation of phagocytic PMNs activity by NPY, SP, and CGRP, the receptors mediating these effects were not identified in our study. However, the expression of SP (NK_1_, NK_2_, NK_3_) and NPY (Y1, Y2, Y4, Y5), membrane specific receptors on human PMNs, was previously confirmed by both molecular and pharmacological approach. Furthermore, it has been shown that SP and NPY had regulatory effect on human PMNs via the set of above-mentioned receptors [8,57]. It is difficult to explain why the stimulatory phagocytic effect of NPs was exerted during the opsonin-dependent uptake as well as the opsonin-independent ROS response. The underlying mechanisms for these contradictory effects were not further explored in this study. At present, we can only speculate that the IgG-FcγR interaction favors signals triggered by NPs, whereas CEACAM3 (the opsonin-independent phagocytic receptor that functions specifically in the recognition and clearance of *M. catarrhalis* and nontypable *H. influenzae* by human granulocytes [58]) as well as other innate receptors such as TLR4, exert the opposite effect. Receptors often function cooperatively during microbial uptake and many parallel signaling pathways are simultaneously activated [59]. Furthermore, because signaling during phagocytosis may vary in response to signals from other receptors, it can modify the activation state of the phagocyte [60]. We postulate from this finding that even if a successful engulfment of mucosal pathogens by PMNs involves distinct types of pathogen recognition, the opsonin-dependent pathway seems to be the most relevant in view of the enhanced uptake elicited by neuropeptides. Our results are in partial agreement with the previous data on stimulatory action of NPY against *E. coli*, although it was tested only in opsonic conditions [8].

Unlike the confirmed presence of SP and NPY receptors on PMNs, the data from the literature do not support the presence of functional CGRP-specific receptors on mature granulocytes [61]. Thus, the fact that both critical phagocytic steps of PMNs, namely the engulfment and the ROS generation, were considerably intensified in the presence of CGRP was an unexpected finding. A lack of functional CGRP receptors on the surface of PMNs does not preclude the involvement of other mechanism of CGRP action such as non-specific receptor usage. It was demonstrated that CGRP may enhance phagocytosis as a result of the engagement of phagocytic mannose-receptors [46]. A direct activation of PMNs by CGRP has been additionally reported [62]. Further studies are necessary to find out what mechanisms underline the CGRP-mediated stimulation of PMN phagocytosis. Summing up, the contradictory action of NPs on *M. catarrhalis* and *H. influenzae* engulfment and ROS response by human PMNs seems to be determined both by the nature of pathogen recognition and by the physiological status of PMN donor. This type of bimodal effect has been reported for various neuropeptides as well as various concentrations of the same neuropeptide on phagocytosis, reactive oxygen production, phagocytic cell adherence and migration or cytokine release [8,63].

In conclusion, we established a direct antibacterial action of CGRP, NPY, SP, and SOM against *M. catarrhalis* and nontypeable *H. influenzae* as well as (apart from SOM) indirect antibacterial effect of the neuropeptides through immunomodulation of PMN phagocytosis. Therefore, CGRP, NPY, and SP, seem to be important molecules involved in innate host defense against *M. catarrhalis* and in part also against nontypeable *H. influenzae*. In higher concentrations they are bactericidal, whereas in lower, physiologically relevant doses, they show immunomodulating properties. The finding regarding the relevance of 10^−8^ M and lower NPs doses may have. physiological or clinical implications. Substance P, NPY, and CGRP may induce chemotaxis of neutrophils, monocytes and macrophages [64,65], therefore attract phagocytic cells to the site of infection. The NPY can modulate critical phagocytic function of PMNs including engulfment and ROS production [8]. Substance P may significantly induce an expression of cyclooxygenase-2, an activation of nuclear factor NF-κβ, and release of prostaglandin PGE_2_ from PMNs [57], as well as trigger mast cells priming and degranulation [66,67], all leading to an inflammatory response. In the context of adaptive immunity, the SP-mediated release of IgG1, IgG3, and IgG4 subclasses implicates its possible importance in antibody-mediated responses during infection [68]. Furthermore, CGRP, NPY, SP, and SOM can also directly affect T cell functions, as they can modulate T cell migration [69] or induce secretion of cytokines such as IL-2, IFN-γ, IL4, and IL-10 by Th1 and Th2 cells in response to antigenic stimulation [3]. All these events may contribute to the local antimicrobial activity of neuropeptides *in vivo*.

It is noteworthy that low NP levels may act in concert with other inflammatory mediators. Such synerginistic response of low SP doses under stimulation with LPS has been shown to potentiate the neutrophil adherence to epithelial cells and cytokine release [70]. NPY and SP can also activate epithelial cells to produce other antimicrobials such as cathelicidins or β-defensins [2] whose bactericidal activities against *M. catarrhalis* and nontypeable *H. influenzae* have been documented [71]. Our observations expand on the previous findings for NPY, CGRP, SP, and are new in the case of SOM direct involvement in antibacterial innate immunity. They show also that neuropeptide-dependent modulation of phagocytosis of the respiratory pathogens under study is a bidirectional phenomenon. However, further research is needed to provide the insight into the particular mechanisms of NPs action and their real clinical significance in the microbial diseases caused by *M. catarrhalis* and *H. influenzae*.

## Conclusion

The present results indicate that neuropeptides such as CGRP, NPY, and SP can effectively participate in the direct and indirect (immunomodulating) elimination of human-specific respiratory pathogens and seem to be important molecules involved in the innate host defense against *M. catarrhalis* and nontypeable *H. influenzae*.

## Abbreviations

AUC_CL_: area under chemiluminescence curve; CL: luminol-dependent chemiluminescence assay; CGRP: calcitonin gene related peptide; FITC: fluorescein isothiocyanate; fMLF: N-formyl-L-methionyl-L-leucyl-L-phenylalanine; Mc: *Moraxella catarrhalis*; MFI: mean fluorescence intensity; MIC: minimal inhibitory concentration; NP: neuropeptide; NPY: neuropeptide Y; NTHi: nontypeable *Haemophilus influenzae*; ONPG: *o*-nitrophenyl-β-D-galactopyranosid; PH_CL_: peak height of chemiluminescence curve; PMA: phorbol myristate acetate; PMNs: polymorphonuclear leukocytes; RLU: relative light unit; ROS: reactive oxygen species; SOM: somatostatin; SP: substance P.

## Competing interest

The authors declare that there are no conflicts of interest.

## Authors’ contributions

DA conceived the study, conducted the majority of experimental work, performed statistical analyses, interpreted the data and wrote the manuscript. AJ conceived the study and helped to finalize the manuscript. PM performed analyses on relationships between activities of neuropeptides and their physicochemical characterization and co-wrote the manuscript. AS performed β-galactosidase assays and in part radial diffusion assays. JG provided expert advice with regards to membrane-peptide interaction. ZD-K identified clinical isolates and provided expert advice regarding antimicrobial assays. All authors read and approved the final manuscript.
